# Cash interventions to improve clinical outcomes for pulmonary tuberculosis: systematic review and meta-analysis

**DOI:** 10.2471/BLT.18.208959

**Published:** 2018-06-04

**Authors:** Aaron Richterman, Jonathan Steer-Massaro, Jana Jarolimova, Liem Binh Luong Nguyen, Jennifer Werdenberg, Louise C Ivers

**Affiliations:** a Department of Medicine, Brigham and Women’s Hospital, 75 Francis Street, Boston, MA 02115, United States of America (USA).; b Department of Obstetrics and Gynecology, Boston University School of Medicine, Boston, USA.; c Department of Medicine, Massachusetts General Hospital, Boston, USA.; d Infection, Antimicrobials, Modelling and Evolution, Unité Mixte de Recherche 1137, INSERM, Paris, France.; e Department of Pediatrics, Dell Children’s Hospital, Austin, USA.; f Center for Global Health, Massachusetts General Hospital, Boston, USA.

## Abstract

**Objective:**

To assess cash transfer interventions for improving treatment outcomes of active pulmonary tuberculosis in low- and middle-income countries.

**Methods:**

We searched PubMed®, Embase®, Cochrane Library and ClinicalTrials.gov for studies published until 4 August 2017 that reported on cash transfer interventions during the treatment of active pulmonary tuberculosis in low- and middle-income countries. Our primary outcome was a positive clinical outcome, defined as treatment success, treatment completion or microbiologic cure. Using the purchasing power parity conversion factor, we converted the amount of cash received per patient within each study into international dollars (Int$). We calculated odds ratio (OR) for the primary outcome using a random effects meta-analysis.

**Findings:**

Eight studies met eligibility criteria for review inclusion. Seven studies assessed a tuberculosis-specific intervention, with average amount of cash ranging from Int$ 193–858. One study assessed a tuberculosis-sensitive intervention, with average amount of Int$ 101. Four studies included non-cash co-interventions. All studies showed better primary outcome for the intervention group than the control group. After excluding three studies with high risk of bias, patients receiving tuberculosis-specific cash transfer were more likely to have a positive clinical outcome than patients in the control groups (OR: 1.77; 95% confidence interval: 1.57–2.01).

**Conclusion:**

The evidence available suggests that patients in low- and middle-income countries receiving cash during treatment for active pulmonary tuberculosis are more likely to have a positive clinical outcome. These findings support the incorporation of cash transfer interventions into social protection schemes within tuberculosis treatment programmes.

## Résumé

**Objectif:**

Évaluer le rôle des interventions de transfert de fonds pour améliorer les résultats du traitement de la tuberculose pulmonaire active dans les pays à revenu faible et intermédiaire.

**Méthodes:**

Nous avons recherché, dans les bases de données de PubMed®, d'Embase®, de ClinicalTrials.gov et de la Cochrane Library, des études publiées jusqu'au 4 août 2017 qui mentionnaient des interventions de transfert de fonds durant le traitement de la tuberculose pulmonaire active dans des pays à revenu faible et intermédiaire. Le critère principal pris en compte était l'obtention d'un résultat clinique positif, défini comme la réussite du traitement, l'achèvement du traitement ou la guérison microbiologique. À l'aide du taux de conversion en parité de pouvoir d'achat, nous avons converti le montant des fonds versés par patient dans chaque étude en dollars internationaux ($ int.). Nous avons calculé le rapport des cotes (RC) pour le critère principal au moyen d'une méta-analyse à effets aléatoires.

**Résultats:**

Huit études remplissaient les critères d'admissibilité permettant d'être incluses dans notre revue. Sept études évaluaient une intervention spécifique à la tuberculose, avec un montant moyen versé compris entre 193 et 858 $ int. Une étude évaluait une intervention prenant en compte la tuberculose, avec un montant moyen de 101 $ int. Quatre études comprenaient des co-interventions non financières. Toutes les études montraient un critère principal plus souvent atteint dans le groupe expérimental que dans le groupe témoin. Après avoir exclu trois études qui présentaient un risque de biais important, il est ressorti que les patients qui bénéficiaient de transferts de fonds spécifiques à la tuberculose étaient plus susceptibles d'avoir un résultat clinique positif que les patients des groupes témoins (RC: 1,77; intervalle de confiance de 95%: 1,57-2,01).

**Conclusion:**

Les données disponibles suggèrent que les patients des pays à revenu faible et intermédiaire qui reçoivent des sommes d'argent durant leur traitement contre la tuberculose pulmonaire active sont plus susceptibles d'avoir un résultat clinique positif. Ces conclusions vont dans le sens de l'intégration d'interventions de transfert de fonds dans les plans de protection sociale des programmes de traitement de la tuberculose.

## Resumen

**Objetivo:**

Evaluar las intervenciones de transferencias de efectivo para mejorar los resultados del tratamiento de la tuberculosis pulmonar activa en los países con ingresos entre bajos y medios.

**Métodos:**

Se realizaron búsquedas en PubMed®, Embase®, Cochrane Library y ClinicalTrials.gov en busca de estudios publicados hasta el 4 de agosto de 2017 que informaran sobre intervenciones de transferencias de efectivo durante el tratamiento de la tuberculosis pulmonar activa en países con ingresos entre bajos y medios. El resultado principal fue un resultado clínico positivo, definido como éxito del tratamiento, finalización del tratamiento o curación microbiológica. Con el factor de conversión de paridad del poder adquisitivo, se convirtió la cantidad de dinero en efectivo recibido por paciente dentro de cada estudio en dólares internacionales. Se calculó el cociente de posibilidades (CP) para el resultado principal mediante un metanálisis de efectos aleatorios.

**Resultados:**

Ocho estudios cumplieron los criterios de elegibilidad para ser incluidos en la revisión. Siete estudios evaluaron una intervención específica para la tuberculosis, con una cantidad promedio de efectivo que osciló entre 193 e 858 dólares internacionales. Un estudio evaluó una intervención sensible a la tuberculosis, con una cantidad promedio de 101 dólares internacionales. Cuatro estudios incluían cointervenciones no monetarias. Todos los estudios mostraron mejores resultados principales para el grupo de intervención que para el grupo control. Después de excluir tres estudios con alto riesgo de sesgo, los pacientes que recibieron una transferencia de efectivo específica para la tuberculosis tuvieron mayores probabilidades de obtener un resultado clínico positivo que los pacientes de los grupos control (CP: 1,77; intervalo de confianza (IC) del 95%: 1,57 a 2,01).

**Conclusión:**

Las pruebas disponibles indican que los pacientes de países con ingresos entre bajos y medios que reciben dinero en efectivo durante el tratamiento de la tuberculosis pulmonar activa tienen más probabilidades de obtener un resultado clínico positivo. Estas conclusiones apoyan la incorporación de las intervenciones de transferencias de efectivo en los planes de protección social dentro de los programas para el tratamiento de la tuberculosis.

## ملخص

**الغرض:**

تقييم تدخلات التحويلات النقدية الساعية إلى تحسين نتائج علاج السل الرئوي النشط في البلدان ذات الدخل المنخفض والمتوسط.

**الطريقة:**

لقد قمنا بالبحث في كل من PubMed®، وEmbase®، ومكتبة Cochrane Library، وClinicalTrials.gov، عن الدراسات المنشورة حتى 4 أغسطس/آب 2017، والتي تناولت تدخلات التحويلات النقدية أثناء علاج السل الرئوي النشط في البلدان ذات الدخل المنخفض والمتوسط. وكانت نتائجنا الأولية نتيجة سريرية إيجابية، تم تعريفها بأنها نجاح العلاج، أو إكمال العلاج، أو التعافي الميكروبيولوجي. وباستخدام معامل تحويل تعادل القوة الشرائية، قمنا بتحويل المبلغ النقدي المستلم لكل مريض في كل دراسة إلى دولار دولي. حسبنا نسبة الاحتمال (OR) للنتائج الأولية باستخدام التحليل التلوي العشوائي.

**النتائج:**

توافقت ثماني دراسات مع معايير الأهلية لتضمينها في المراجعة. قامت سبع دراسات بتقييم التدخل الخاص بمرض السل، مع متوسط مبلغ نقدي يتراوح من 193 إلى 858 دولاراً دولياً. وقيمت إحدى الدراسات التدخل الحساس لمرض السل، بمتوسط مبلغ قدره 101 دولاراً دولياً. شملت أربع دراسات التدخلات غير النقدية المشتركة. أظهرت جميع الدراسات نتائج أولية أفضل لمجموعة التدخل من المجموعة المرجعية. بعد استبعاد ثلاث دراسات ذات خطورة عالية للتحيز، كان المرضى الذين يتلقون تحويلات نقدية خاصة بالسل أكثر عرضة لتحقيق نتائج سريرية إيجابية من المرضى في المجموعات المرجعية (نسبة الأرجحية: 1.77؛ فاصل الثقة 95٪: من 1.57 إلى 2.01).

**الاستنتاج:**

تشير الأدلة المتاحة إلى أن المرضى في البلدان ذات الدخل المنخفض والمتوسط ، الذين يتلقون مبالغ نقدية أثناء علاجهم من مرض السل الرئوي النشط، هم أكثر عرضة لتحقيق نتائج سريرية إيجابية. تدعم هذه النتائج الاتجاه لدمج تدخلات التحويلات النقدية في نظم الحماية الاجتماعية ضمن برامج علاج مرض السل.

## 摘要

**目的:**

评估通过现金转移干预措施以改善中低收入国家活动性肺结核的治疗效果。

**方法:**

在 PubMed®、Embase®、考克兰图书馆和美国临床试验数据库 (ClinicalTrials.gov) 搜索截至 2017 年 8 月 4 日发表的研究时，我们发现了报道的中低收入国家活动性肺结核治疗期间的现金转移干预。我们的主要成果是积极的临床疗效，因此而被定义为治疗成功、治疗完成或微生物学治疗。通过使用购买力平价换算，我们将每项研究中每名患者接收的现金数额换算成国际美元 (Int$)。我们采用随机效应荟萃分析法为主要结果计算了优势比。

**结果:**

其中，有八项研究符合纳入审查的资格标准。有七项研究评估了结核病专项干预，其平均现金数额从 193 至 858 国际美元不等。有一项研究评估了结核病敏感干预，其平均现金数额为 101 国际美元。有四项研究包含非现金共同干预。所有研究均表明干预组的主要结果优于对照组。通过排除三项具有高度偏见风险的研究，接收结核病专项现金转移的患者比控制组中的患者更可能出现积极的临床疗效（或：1.77；95% 置信区间：1.57–2.01)。

**结论:**

有效证据显示中低收入国家的患者在治疗活动性肺结核期间接收现金干预更有可能拥有积极的临床疗效。这些发现支持将结核病治疗方案中的现金转移干预纳入社会保障计划中。****

## Резюме

**Цель:**

Оценить влияние финансовой помощи на улучшение результатов лечения активного туберкулеза легких в странах с низким и средним уровнем дохода.

**Методы:**

Авторы провели поиск в базах данных PubMed®, Embase®, Кохрановской библиотеке (Cochrane Library) и в реестре ClinicalTrials.gov на предмет исследований, опубликованных до 4 августа 2017 года, в которых сообщалось об оказании финансовой помощи в ходе лечения активного туберкулеза легких в странах с низким и средним уровнем дохода. В качестве основного результата рассматривался положительный клинический исход, определяемый как эффективное лечение, завершение лечения или микробиологическое излечение. Используя коэффициент пересчета паритета покупательной способности, авторы перевели количество денежных средств, полученных каждым пациентом в рамках каждого исследования, в международные доллары. Авторы рассчитали отношение шансов (ОШ) для основного результата, используя метаанализ случайных эффектов.

**Результаты:**

Восемь исследований соответствовали критериям приемлемости для включения в обзор. В семи исследованиях оценивалась финансовая помощь, связанная с лечением туберкулеза, со средним количеством денежных средств в диапазоне от 193 до 858 международных долларов. В одном исследовании оценивалась финансовая помощь, связанная с лечением туберкулеза, со средним количеством денежных средств, равным 101 международному доллару. Четыре исследования включали неденежные совместные вмешательства. Во всех исследованиях для группы вмешательства наблюдался лучший основной результат по сравнению с контрольной группой. После исключения трех исследований с высоким риском систематической ошибки было обнаружено, что у пациентов, получающих финансовую помощь, связанную с лечением туберкулеза, чаще наблюдался положительный клинический исход, чем у пациентов в контрольных группах (ОШ: 1,77; 95%-й ДИ: 1,57–2,01).

**Вывод:**

Имеющиеся данные свидетельствуют о том, что у пациентов в странах с низким и средним уровнем дохода, получающих финансовую помощь во время лечения активного туберкулеза легких, чаще наблюдается положительный клинический исход. Эти результаты подтверждают рациональность включения финансовой помощи в схемы социальной защиты в рамках программ лечения туберкулеза.

## Introduction

Tuberculosis remains one of the top 10 causes of death worldwide, with the highest burden of disease in low- and middle-income countries.^1^ In these countries, the disease disproportionately affects the most vulnerable populations.^1^^,^^2^

In 2015, the World Health Organization’s (WHO’s) End TB Strategy set the goal of a 90% reduction in tuberculosis deaths, an 80% reduction in tuberculosis incidence rate and zero catastrophic costs for tuberculosis-affected families by 2030.^3^ These goals explicitly acknowledge the need to both directly treat people infected with the disease and address social determinants of health to improve tuberculosis outcomes.

Social protection policies protect individuals or households during periods when they are unable to financially support themselves because of a range of conditions, such as illness or disability.^4^ Cash transfer interventions, defined as cash payments provided to selected beneficiaries by formal institutions, are one form of social protection that has been proposed in the setting of tuberculosis.^5^^,^^6^ Such interventions can either be tuberculosis-specific or tuberculosis-sensitive.^6^ Tuberculosis-specific interventions target directly tuberculosis patients and their households, and are typically incorporated into existing tuberculosis treatment programmes.^6^ A tuberculosis-sensitive intervention is part of a broader social protection scheme, potentially affecting tuberculosis outcomes by targeting communities and groups that are at high risk for tuberculosis. The effect on health outcomes, cost–effectiveness and feasibility of these two strategies are not well established and likely to vary based on the local social protection and health-care infrastructure.

Since a review in 2011 on the effects of cash transfer interventions on tuberculosis outcomes in low- and middle-income countries was inconclusive,^7^ we assessed the current state of the evidence for such interventions. We were especially interested if cash transfer to people receiving treatment for active pulmonary tuberculosis affects their clinical outcomes.

## Methods

We followed the Preferred Reporting Items for Systematic Reviews and Meta-Analyses guidelines.^8^ The review protocol is available from the corresponding author.

To identify studies on the use of cash transfer interventions during the treatment of active pulmonary tuberculosis in low- and middle-income countries, we searched the online databases PubMed®, Embase®, Cochrane Library and ClinicalTrials.gov. We used the search string “Tuberculosis” AND (“financial support” OR “token economy” OR “reimbursement” OR “economic burden” OR “incentives” OR “cash transfer” OR “enablers”) to identify studies published between the databases’ inceptions and 4 August 2017. We also manually reviewed reference lists of identified systematic reviews, relevant articles and abstracts from the Union World Conference on Lung Health 2011–2016.

### Eligibility criteria

We considered clinical trials and observational studies published in English, Spanish or French that assessed cash transfer interventions directed at people initiating treatment for microbiologically confirmed or clinically suspected active pulmonary tuberculosis. We used the WHO definition for tuberculosis and the 2017 World Bank’s classification of low- and middle-income countries.^9^^,^^10^ We included studies that reported standard outcomes of treatment completion, microbiologic cure or treatment success, which includes both treatment completion and cure.^10^

### Study selection and data collection

After removing duplicate records, two reviewers independently screened titles and abstracts of all records for inclusion in full-text review. After screening, two different reviewers independently applied eligibility criteria to each full-text article. Two reviewers then proceeded to data extraction using a standardized form created for the study (Box 1). Disagreements were settled by consensus among all authors.

Box 1 **Type of data extracted from identified studies on cash interventions to improve tuberculosis outcome**

We extracted data on location; urban and rural setting; time frame; study design; number of subjects; age and gender of participants; HIV prevalence; number with microbiologically confirmed tuberculosis; number with confirmed or suspected MDR and XDR tuberculosis; type of usual care for tuberculosis; annual individual or household income; whether the intervention was conditional; tuberculosis-specific or sensitive intervention; concurrently implemented co-interventions; primary and secondary outcomes.

HIV: human immunodeficiency virus; MDR: multidrug resistant; XRT: extensively-drug resistant.

To better understand the relative amount of cash distributed in the included studies, we converted the average and maximum possible amount of cash received per patient within each study into international dollars (Int$) using the purchasing power parity conversion factor, and then adjusted for inflation into 2016 Int$ with the local inflation conversion factor.^11^ If the average amount of cash received by patients in the intervention group was not reported in the article, we contacted the authors to provide the figures.

Because tuberculosis disproportionately affects the poorest households within a given context,^12^ we estimated the average amount of cash received per patient as a proportion of annual individual income by dividing the average amount of cash received per patient by the median income per capita of the lowest quintile of that country from the time period of the study.^11^ Household-level income data were not available to estimate the interventions as proportion of annual household income.

### Assessment of bias

For the randomized study, we assessed risk of bias using the Cochrane Collaboration Risk of Bias Tool, and defined a randomized study as overall high risk of bias if the trial met criteria for high risk of bias in more than one assessed domain.^13^ We assessed risk of bias within non-randomized studies using the Newcastle-Ottawa Scale, defining a non-randomized study as overall high risk of bias if it had zero stars in any of the three assessed categories.^14^ We generated a funnel plot to evaluate publication bias for studies included in the meta-analysis.

### Data analysis

All identified studies were included in a qualitative synthesis. After excluding studies at overall high risk of bias, we generated summary effect measures using a random effects model for our primary outcome of interest, the odds ratio (OR) of a positive clinical outcome, defined as either a treatment success; treatment completion, if a study did not report treatment success; or microbiologic cure, if a study did not report treatment success or treatment completion. If a study reported ORs adjusted for potential confounders we included these ratios in our analysis. We assessed heterogeneity by using the Cochran’s *Q* test and the *I^2^* statistic.

Among studies included in meta-analysis, we wanted to investigate sources of heterogeneity, including average amount of cash transfer, presence of non-cash co-intervention, treatment success rate in the control group, urban or rural setting, human immunodeficiency (HIV) prevalence, multidrug resistance (MDR) or extensive-drug resistance (XDR) tuberculosis prevalence and World Bank income classification. However, there was not enough information available to complete a random effects meta-regression model using any of these variables.

We used Comprehensive Meta-Analysis software version 3 (Biostat, Inc., Englewood, United States of America) and Review Manager Version 5.3 (The Cochrane Collaboration, London, United Kingdom of Great Britain and Northern Ireland) for data analysis.

## Results

### Study selection

**Fig. 1:**
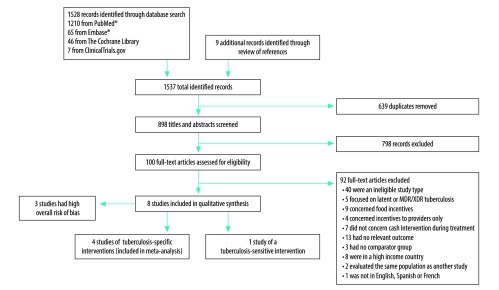
Flowchart showing the selection of studies on cash interventions to improve tuberculosis clinical outcomes, 1991–2017

We identified 1537 publications and after removal of 639 duplicates, we screened 898 titles and abstracts yielding 100 full-text articles to be assessed for eligibility. Of these full-text articles, 92 were excluded (Fig. 1). We included eight eligible articles: one randomized control trial,^15^ two non-randomized intervention studies,^16^^,^^17^ and five observational studies,^18^^–^^22^ comprising a total of 21 976 subjects.

### Study settings and populations

Table 1 summarizes the settings and populations of the included studies. With the exception of one study that took place in 1989–1990,^17^ the studies assessed cash transfer interventions between 2004 and 2015. The settings varied: one study took place in a rural clinic,^17^ one in a large rural secondary-care facility,^16^ four in urban centres,^15^^,^^19^^–^^21^ and two were nation-wide studies.^18^^,^^22^ Three of the studies took place in countries currently on the WHO list of high-burden countries for tuberculosis^16^^,^^19^^,^^22^^,^^23^ and two other studies were in a country currently considered high burden for MDR tuberculosis.^15^^,^^21^

**Table 1:** Design, setting and population of included studies in the systematic review on cash interventions to improve tuberculosis clinical outcomes, 1991–2017

Author, publication year	Year of study	Study design and setting	Usual care	% male	% smear positive	% HIV	% MDR tuberculosis	Intervention group	Control group
**Tuberculosis-specific interventions**									
Farmer et al.,^17^ 1991	1989–1990	Cluster non-randomized intervention study in a clinic in rural Haiti	Free care, no community health workers or DOTS	33	100	5	NR	People with newly diagnosed tuberculosis from sector adjacent to clinic	People with newly diagnosed tuberculosis from outside sector adjacent to clinic
Chirico et al.,^20^ 2011	2004–2008	Retrospective cohort in one health district of Buenos Aires, Argentina	51% of patients receiving DOTS, cost of care NR	57	NR	6	0.91	All people with newly diagnosed tuberculosis reported to national tuberculosis control programme	People with newly diagnosed tuberculosis who did not get the intervention because deemed not to have the financial need, chosen at random among all people who did not get the intervention
Rocha et al.,^21^ 2011	2007–2010	Cohort with historical control in eight shantytowns in Lima, Peru	DOTS, free care	NR	NR	NR	NR	People with newly diagnosed tuberculosis from households in the national tuberculosis programme where intervention had been implemented	People with newly diagnosed tuberculosis from households in the national tuberculosis programme where the intervention had not yet been implemented
Ciobanu et al.,^18^ 2014	2008, 2011	Nation-wide retrospective cohort with historical control in the Republic of Moldova	DOTS, cost of care NR	69	36	3	0	Adults with drug-susceptible tuberculosis registered for treatment in 2011 (after introduction of incentives)	Adults with drug-susceptible tuberculosis registered for treatment in 2008 (before introduction of incentives)
Lu et al.,^19^ 2015	2006–2010	Retrospective cohort in Shanghai, China	DOTS, free care	63	100	NR	0	Migrants treated for smear-positive pulmonary tuberculosis living in one of the eight districts providing cash	Migrants treated for smear-positive pulmonary tuberculosis living in one of the nine districts not providing cash
Ukwaja et al.,^16^ 2017	2014	Prospective pre- and post- intervention in a large, rural, secondary-care facility in Ebonyi State, Nigeria	DOTS, cost of care NR	54	55	15	0	All registered people receiving first-line anti-tuberculosis treatment at study site during 3-month period of intervention	All registered people receiving first-line anti-tuberculosis treatment at study site during 3-month period without financial package
Wingfield et al.,^15^ 2017	2014–2015	Cluster randomized control trial in thirty-two contiguous shantytowns in Callao, Peru	DOTS, free care	62	70	5	9	People starting treatment for tuberculosis administered by the national tuberculosis programme, randomized to receive the socioeconomic support intervention	People starting treatment for tuberculosis administered by the national tuberculosis programme, randomized not to receive the socioeconomic support intervention
**Tuberculosis-sensitive interventions**									
Torrens et al.,^22^ 2016	2010	Nation-wide retrospective cohort in Brazil	Free diagnostics and treatment for all patients. Tuberculosis patients only enrolled into directly observed therapy if judged to be able to complete treatment	50	NR	7	0	People with newly diagnosed non-MDR tuberculosis recorded in the national database who received cash during treatment	People with newly diagnosed non-MDR tuberculosis recorded in the national database who were eligible for cash interventions, but only started to receive them after treatment due to administrative delays

DOTS: directly observed therapy, short course; HIV: human immunodeficiency virus; MDR: multidrug resistant; NR: not reported.

One study focused on migrant workers, a high-risk group within an urban centre.^19^ The remaining studies evaluated all tuberculosis patients identified within a given geographic or clinical service area. The control groups were either patients randomized to the non-intervention group,^15^ living in a non-intervention area,^17^^,^^19^ historical controls from the same population before the implementation of the intervention,^16^^,^^18^^,^^21^ eligible for the intervention, but not yet receiving cash, because of administrative delay,^22^ or not eligible for the intervention, because of insufficient financial need.^20^

Prevalence of HIV seropositivity among the study populations was 0–15% in the six studies reporting the outcome.^15^^,16.14,^^17^^,^^18^^,^^22^ Patients with MDR/XDR tuberculosis were excluded from four studies,^16^^,^^18^^,^^19^^,^^22^ two studies reported low prevalence (1–9%),^15^^,^^20^ while two did not report on drug susceptibility.^17^^,^^21^ Five studies reported free care for tuberculosis,^15^^,^^17^^,^^19^^,^^21^^,^^22^ with the others not specifically commenting on the cost of care.^16^^,^^18^^,^^20^ Participants in six studies received the WHO recommended directly observed therapy, short-course.^24^

### Tuberculosis-specific interventions

In total, seven studies evaluated tuberculosis-specific cash transfer interventions (Table 2).^15^^–^^21^ Six of these studies were at least partially conditional on clinic attendance or treatment completion,^15^^–^^20^ and one did not report whether the intervention was conditional.^21^ Four studies described an additional transportation reimbursement.^17^^–^^19^^,^^21^

**Table 2:** Type of cash transfer intervention of included studies in the systematic review on cash interventions to improve tuberculosis clinical outcomes, 1991–2017

Author, year	Cash transfer intervention	Conditional intervention; method of cash delivery	Maximum cash, Int$^a^	Average cash, Int$^a^	Average cash as percent of annual income^b^	Additional interventions^c^
**Tuberculosis-specific interventions**						
Farmer et al.,^17^ 1991	Monthly cash transfer and travel reimbursement	Mixed: travel reimbursement conditional on clinic attendance, monthly transfer not conditional, because clinic staff would come to the homes of the patients missing clinic visits; cash	900	900	173	Daily visits by community health worker during first month. Food supplements for first 3 months. If the patient did not attend the appointment, someone from the clinic went to the household to investigate
Chirico et al.,^20^ 2011	Monthly cash during period of treatment equal to low civil service salary. For patients not otherwise protected by other social safety net benefits	Yes: clinic visits; cash delivered by the bank employee after the patient presented documentation of programme enrolment	NA	NA	NA	None
Rocha et al.,^21^ 2011	Cash transfers for transportation, poverty reduction, and other tuberculosis-associated costs	NR	NA	291	17 (5.5)^d^	Microcredit loans, vocational training, microenterprise activities (e.g. raising animals, home-based manufacturing), food transfers, home visits, community workshops, psychological assessment
Ciobanu et al.,^18^ 2014	Combination of smaller monthly cash, larger cash at treatment completion, and variable transport reimbursement	Yes: clinic visits and/or treatment completion; NR	773	489	20	Vouchers for food/hygiene products, other support (clothes, wood for cooking). Provided to only a subset of the intervention group
Lu et al.,^19^ 2015	Monthly cash transfer and transportation subsidy	Yes: clinic visits; cash delivered by the programme staff at the community health centre or district centre for disease control	253	NA	NA	None
Ukwaja et al.,^16^ 2017	Monthly cash transfer equivalent to median direct cost for tuberculosis care. Appointments for tuberculosis patients receiving cash arranged to not coincide with the control group	Yes: clinic visits; cash delivered at the clinic by the trained staff member	193	193	11	None
Wingfield et al.,^15^ 2017	Cash transfers throughout treatment to defray average household tuberculosis-associated costs, estimated to be 10% annual household income in this setting	Yes: details unspecified; deposit into bank account	436	355	13 (3.6)^d^	Household visits with education on tuberculosis transmission, treatment, and preventive therapy and on household finances. Community meetings for information, support, empowerment and stigma reduction
**Tuberculosis-sensitive interventions**						
Torrens et al.,^22^ 2016	Monthly cash to female head of household as part of *Bolsa Familia* programme	Yes: 1) Attendance at prenatal, postnatal monitoring sessions 2) Nutrition and vaccine monitoring for children 3) School attendance; Withdrawal using designated debit card distributed by programme	222	101	3.1	None

Int$: international dollars; NA: not available; NR: not reported.

^a^ We converted the average amount of cash received per patient into Int$ purchasing power parity conversion factor, and then adjusted for inflation into 2016 dollars with the local inflation conversion factor.^11^

^b^ Estimated percentage of annual individual income, unless otherwise specified

^c^ Additional interventions did not involve cash.

^d^ Reported percentage of annual household income.

Four studies did not report the average amount of cash received by patients in the intervention group. We contacted the authors of these studies and authors of two studies provided the amount,^16^^,^^18^ while this information was not available for other studies.^19^^,^^20^ The average amount of cash distributed ranged from Int$ 193–858. Two studies chose the amount of cash based on previous work estimating local tuberculosis-associated household costs,^15^^,^^16^ including the CRESIPT project in Peru, the only identified randomized control trial.^15^ The CRESIPT project distributed cash using bank deposit (hypothesizing that opening a bank account was empowering to the study subjects),^15^ whereas other studies used actual cash^16^^,^^17^^,^^19^^,^^20^ or did not report method of delivery.^18^^,^^21^ Four studies included some additional non-cash co-intervention, including home visits, community meetings, food vouchers and psychological intervention.^15^^,^^17^^,^^18^^,^^21^

### Tuberculosis-sensitive interventions

Only one study described a tuberculosis-sensitive intervention, a nation-wide retrospective cohort study in Brazil of tuberculosis patients in the *Bolsa Familia* programme. The programme is a monthly cash transfer to poor people that is conditional on attending antenatal care, nutrition and vaccine monitoring for their children and that their young children attend school.^22^ People with newly diagnosed non-MDR tuberculosis who received cash during treatment were compared to those who were eligible for cash at the time of treatment, but did not receive it, because of administrative delays. The average amount of total cash delivered to the intervention group was Int$ 101, representing an estimated 3.1% of annual individual income. Cash could be claimed by the patient monthly using a designated bank card.

### Outcomes

Most studies (5) reported the primary outcome of treatment success,^15^^,^^16^^,^^18^^–^^20^ one reported treatment completion^21^ and two reported microbiologic cure (Table 3).^17^^,^^22^ Four studies controlled for potential confounders.^16^^,^^18^^,^^19^^,^^22^ Two of the three studies that reported loss to follow-up found significantly less loss to follow-up in the intervention group.^15^^,^^16^^,^^18^ Of the four studies which reported mortality, none found a difference between the intervention and control groups.^15^^–^^18^

**Table 3:** Outcomes of included studies in the systematic review on cash interventions to improve tuberculosis clinical outcomes, 1991–2017

Author, year	Primary outcome								
	Outcome indicator^a^	Sample size			No. patients of with primary outcome		OR (95% CI)	Adjusted covariates	Secondary outcomes (intervention versus control)
		Intervention	Control	Intervention		Control			
**Tuberculosis-specific interventions**									
Farmer et al.,^17^ 1991	Microbiologic cure	30	30	30		13	79.08 (4.42–1 413.33)	None	Sputum positivity at 6 months (0% vs 13%); pulmonary symptoms at 1 year (7% vs 43%); weight gained during first year (10.4 lbs vs 1.7 lbs); return to work after 1 year (93% vs 47%); 18-month mortality (0% vs 10%)
Chirico et al.,^20^ 2011	Treatment success	804	847	750		666	1.19 (1.03–1.37)	None	None
Rocha et al.,^21^ 2011	Treatment completion	307	1554	298		1414	3.28 (1.65–6.51)	None	Health insurance registration (98% vs 36%); contact screening (96% vs 82%); rapid MDR-tuberculosis testing (92% vs 67%); HIV testing (97% vs 31%); contact preventive therapy initiation (88% vs 39%) and completion (87% vs 27%)
Ciobanu et al.,^18^ 2014	Treatment success	2378	2492	2081		1964	2.00 (1.61–2.22)^b^	Place of residence, sex, age, occupation, homelessness, HIV, type of tuberculosis	Treatment failure (2% vs 5%); loss to follow-up (5% vs 10%); death (5% vs 6%)
Lu et al.,^19^ 2015	Treatment success	3290	2413	NR		NR	1.65 (1.40–1.95)^b^	Gender, age, occupation, per capita GDP of district, density of population, tuberculosis specialists per 100 patients	None
Ukwaja et al.,^16^ 2017	Treatment success	121	173	104		123	2.30 (1.20–4.30)^b^	Sex, age, rural/urban residence, new/previously treated tuberculosis, HIV, smear-positivity	Loss to follow-up (5% vs 20%); transferred out (1% vs 0%); death (7% vs 6%); smear negative at 2 months (88 vs 92)
Wingfield et al.,^15^ 2017	Treatment success	135	147	87		78	1.60 (0.99–2.59)	None	Loss to follow-up (16% vs 18%); death (4% vs 4%)
**Tuberculosis-sensitive interventions**									
Torrens et al.,^22^ 2016	Microbiologic cure	5788	1467	4752		1128	1.07 (1.04–1.11)^b^	Age, ethnicity, diabetes mellitus, HIV, extrapulmonary tuberculosis, self-administered treatment, rural area, number of rooms in house, inappropriate floor material, baseline household monthly per capita income < US$20, illiteracy	None

CI: confidence interval; GDP: gross domestic product; HIV: human immunodeficiency virus; lbs: pounds; MDR: multidrug resistant; NR: not reported; OR: odds ratio; US$: United States dollars.

^a^ The definitions of the outcomes were: treatment success was positive clinical outcome; treatment completion was if a study did not report treatment success; and microbiologic cure was if a study did not report treatment success or treatment completion.

^b^ Derived from multivariable regression models.

### Bias

**Fig. 2:**
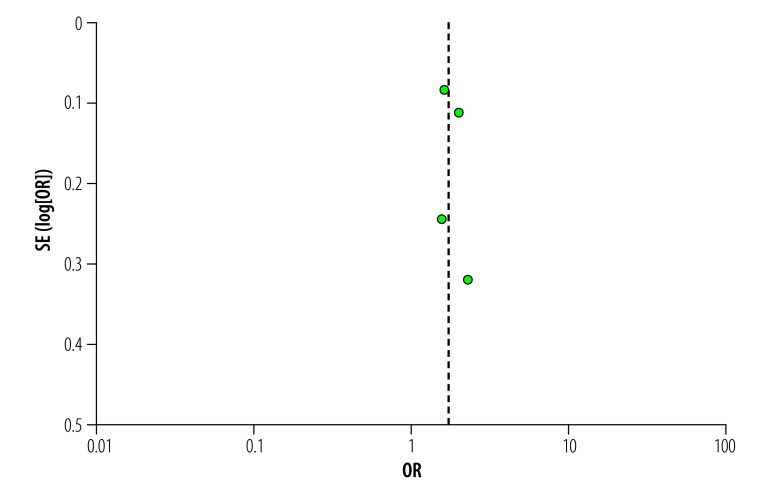
Publication bias of studies included in the meta-analysis on cash interventions to improve tuberculosis clinical outcomes, 1991–2017

OR: odds ratio; SE: standard error.

Note: The dashed vertical line represents the summary OR generated through random effects meta-analysis.

Table 4 shows the risk of bias within individual observational studies and three studies met criteria for high overall risk of bias.^17^^,^^20^^,^^21^ We deemed the randomized control trial^15^ not to have a high overall risk of bias, as only the domain attrition showed high risk: 37% (105/282) of patients were lost to follow-up or not evaluated. The other six domains had a low risk. The funnel plot of studies included in meta-analysis did not show evidence of publication bias (Fig. 2).

**Table 4:** Bias within included observational studies in the systematic review on cash interventions to improve tuberculosis clinical outcomes, 1991–2017

Study, year	Category, no. of stars		
	Selection^a^	Comparability^b^	Outcome^c^
Farmer et al.,^17^ 1991	3	0	2
Chirico et al.,^20^ 2011	3	0	1
Rocha et al.,^21^ 2011	2	0	0
Ciobanu et al.,^18^ 2014	3	2	2
Lu et al.,^19^ 2015	3	2	2
Torrens et al.,^22^ 2016	3	2	3
Ukwaja et al.,^16^ 2017	3	2	2

^a^ A study could be awarded a maximum of four stars for this category.

^b^ A study could be awarded a maximum of two stars for this category.

^c^ A study could be awarded a maximum of two stars for this category.

Note: We used Newcastle-Ottawa Scale to assess bias in observational studies. The more stars the study received the lower the risk of bias.

### Summary effect measures

**Fig. 3:**
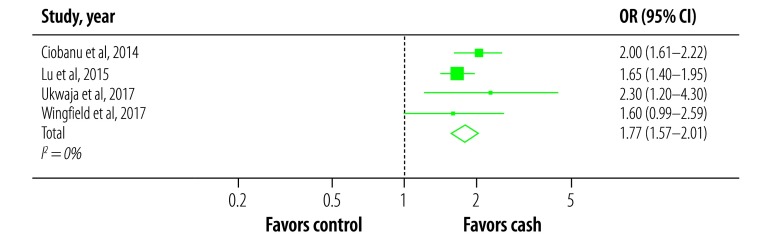
Likelihood of a positive clinical outcome for tuberculosis-specific cash interventions to improve tuberculosis clinical outcomes, 1991–2017

CI: confidence interval; OR: odds ratio.

Note: We excluded three studies with a high overall risk of bias.^17^^,^^20^^,^^21^

Fig. 3 shows the forest plot of the remaining tuberculosis-specific studies after excluding studies at high overall risk of bias. Patients receiving tuberculosis-specific cash transfer were more likely to have a clinical positive outcome than patients in the control groups (OR: 1.77; 95% confidence interval: 1.57–2.01), with *I*^2^ = 0% (*Q* test *P* = 0.44).

## Discussion

The findings of this systematic review and meta-analysis suggest that cash transfer interventions for patients in low- and middle-income countries initiating tuberculosis treatment may improve clinical outcomes. All studies reported improvement in treatment outcomes. However, the overall evidence is weak because we only identified one eligible randomized control trial. Additionally, half of the studies included some non-cash co-intervention and thus some of the positive effects seen may be related to the pooled effects of cash and non-cash interventions.

There are several possible mechanisms by which cash transfer interventions may improve clinical outcomes for tuberculosis patients during treatment.^6^^,^^25^ Both tuberculosis-specific and tuberculosis-sensitive cash transfer interventions can act as direct poverty-reduction measures by offsetting costs caused by the disease. These costs include both direct costs of treatment such as clinic fees, medication costs, travel and/or food, as well as indirect costs incurred through loss of wages. In particular, catastrophic costs, defined as tuberculosis-related costs which exceed 20% of the household’s annual income, have been associated with adverse clinical outcomes.^1^^,^^2^^,^^26^ Two studies have found that, on average, a person with tuberculosis in a low- and middle-income country will experience catastrophic costs as a result of the illness.^2^^,^^27^ In this review, one tuberculosis-specific intervention provided cash equivalent to 173% of estimated annual individual income,^17^ four provided cash equivalent to 10–20% of estimated annual individual income,^15^^,^^16^^,^^18^^,^^21^ and two of these studies also reported the intervention as percentage of annual household income, between 3–6%.^15^^,^^21^ The single tuberculosis-sensitive intervention we identified provided cash equivalent to 3.8% of estimated annual individual income. The difference between tuberculosis-specific and tuberculosis-sensitive interventions may reflect the findings that sensitive interventions are less likely to be effective and affordable by countries for offsetting tuberculosis-associated catastrophic costs than specific interventions.^27^ However, tuberculosis-sensitive interventions also have the advantage of a broader poverty reduction impact, which might improve household economic resilience before a household member develops active tuberculosis infection.

Beyond simply offsetting costs, cash transfer interventions may also serve as an additional incentive for health-seeking behaviour, particularly when distribution is conditional on clinical follow-up or medication adherence.^28^^–^^30^ Several systematic reviews have found a positive effect of conditional cash transfers in low- and middle-income countries on health behaviours and outcomes, including increased use of preventative services, improved childhood nutritional status, decreased self-reported episodes of illness and decreased HIV prevalence.^25^^,^^30^^,^^31^ Another systematic review found that the impact of unconditional cash transfers on health services use and health outcomes was uncertain.^28^ None of the interventions we identified had a completely unconditional cash transfer intervention. The incentive of a conditional intervention may be particularly important in tuberculosis care, where consistent adherence to a multiple-drug regimen for a prolonged treatment course is essential for optimal treatment outcomes. However, a meta-analysis of the effect of incentives and/or enablers on medication adherence in tuberculosis was largely inconclusive, but primarily identified studies in high-income countries, where financial interventions may have less effect.^32^ Tuberculosis-sensitive interventions are likely to lack a tuberculosis-specific incentive, although they may include other conditional elements unrelated to tuberculosis, as in the case of the *Bolsa Familia* programme.^22^

The studies showed substantial heterogeneity in study design. However, there was no measured heterogeneity within the subset of studies with tuberculosis-specific interventions that were not at high overall risk of bias. Although factors related to the population, setting and intervention could cause heterogeneity in the effect size of the interventions, the available information from the limited number of studies did not allow us to determine the impact of these variables.

Whether cash transfers or goods and services, such as direct provision of food, vocational training, psychologic support and housing programmes, are preferable to improve health-related and other outcomes is currently under debate.^33^^–^^35^ A recent meta-analysis found that non-cash socioeconomic interventions, predominantly food provision, may improve clinical outcomes in active tuberculosis.^36^ To better understand which forms of social protection are most effective at improving clinical outcomes for tuberculosis, non-cash strategies should be studied comparatively and in combination with cash transfer interventions.

While beyond the scope of this review, the impact of cash transfer interventions on household and national or subnational outcomes, like contact screening and overall tuberculosis incidence, must also be considered. For example, a multivariable analysis found that municipalities in Brazil with higher coverage by the *Bolsa Familia* programme had a significant reduction in tuberculosis incidence compared to those with lower coverage.^37^

In conclusion, we found some evidence that cash transfer interventions improve treatment outcomes in patients with active pulmonary tuberculosis in low- and middle-income countries, although the overall quality of this evidence is low. These findings support calls by WHO and others to incorporate cash transfer interventions into social protection schemes within tuberculosis treatment programmes.^1^^,^^6^ In addition, high-quality research is needed to better understand the effectiveness of tuberculosis-specific and tuberculosis-sensitive cash transfer interventions, including understanding of the optimal amount, conditional feature, delivery method and implementation strategy.
